# Mercurius Vivus

**Published:** 1873-06

**Authors:** H. L. Ambler


### MERCURIUS VIVUS.

H. L. AMBLER, D. D. S., M. D.

*A continuation of an article in the January No. of the Register.*

Symptoms for the administraton of Mercurius vivus: Tearing, shooting pains in whole side of face and head; teeth are loose and feel to long; generally worse at night in a warm bed worse after exposure to damp or cold air; submaxillary glands are swollen; gums swollen, sensitive, and bleed readily. In neuralgia we have darting, tearing pains on one side, from the temples to the teeth; profuse saliva and perspiration without relief; pain removed by eating, and taking hot or cold substances into the mouth; tongue moist with thick, white coating; sleeplessness; pain may be seated, but frequently shifts from one portion to another of the affected nerve. Dose and repetition should be determined according to the case; from the third to sixth potency in acute, and eighteenth to thirtieth in chronic cases. Some are quite susceptible to its influence; while others are comparatively insensible to medicinal influence, showing neither marked action nor reaction and require low potencies and frequent doses. Generally those where the lymphatic temperament predominates require a dose of one grain once or twice an hour until relieved. Those of highly developed mental organizations are more susceptible to the *action* than reflex action of remedies; thus a higher potency would generally be admissable; but where reaction is well marked, too frequent repetition of dose must be guarded against; also where we have marked *action* and

*reaction* we must be guided by other conditions of the case as to potency and frequency of dose. Those of a bilious temperament are not very receptive to medicinal influence, but reaction being once aroused, it will be powerful and somewhat long; in such cases use the low potencies, and at long intervals. Females generally possess a higher degree of susceptibility than males, thus permitting us to use a high potency, or if a low one at long intervals, about three or five hours; but with them modifying circumstances must be taken into consideration, such as pregnancy, laborious employment, etc. Medicinal influence is more marked upon those living in the country of simple regular habits, where they are not disposed to any regular peculiar dyscrasia, than in towns, and particularly crowded cities, where habits are more artificial, as here the action is more difficult to arouse. The different temperaments of which we have spoken are often found mixed, and habits, modes of living, peculiar idiosyncracies of patients, must not be overlooked in our treatment if we derive the quickest and most favorable results. Do not imagine because you have given a small dose, and your patient improves, that you must hasten and give a larger one; but in the administration of this remedy, if there is no amelioration after using from two to four hours, the frequency of the dose may be desirable; sometimes it is best to cease administration as long as improvement continues, but upon the slightest morbid activity reappearing, to again have recourse to the remedy.

In the long continued exhibition of a remedy the system may become less susceptible to its influence than formerly, then it would be wise to change the potency, or use a remedy similar to the one before used; but should decided improvement follow each dose, then the intervals should be lengthened, thus giving the system a chance to be itself again, leaving the medicinal susceptibility unimpaired until a cure is completed. The remedial properties of this remedy are facts upon which our method of cure is founded; it controls the the formative or crystalizing process of the cell, reaches the center, and changes it by specific action, as it has greater affinity for the disease than the disease has for the parts implicated. These principles are open to the knowledge of all, and being put to the test of daily observation by different ones, there must necessarily be truth in them, otherwise arguments and facts to overthrow them would speedily be brought forward. We have seen much of the mercurius on sale, put up in paste-board boxes; this should not be done as in time it looses its efficiency.

The true dental surgeon never imagines himself perfect in all departments, but welcomes professional light, and displays an interest, from whatever source it may come; nor is he so contracted in mind as to suppose that the arts and sciences which his ancestors practiced were so near perfection that all remaining for him is to follow their directions, and not exercise his own industry or skill trying to improve. Let Onward be the watchword inscribed on humanity's banner as it is borne upon the breeze which cheers on this ever varying life; progress, is God's eternal law. Let not the traditions of past ages, but the noble and exalted hopes of the future stimulate us to greater exertion; being banded together for the one purpose of redeeming suffering fellow creatures from the genius of barbarous treatment; with light and love for our calling, we may be guided to an exalted manhood.

